# Assessment of Platelet Storage Lesions, Viability, and Function in Canine Platelet Concentrate Units Stored at 4°C for 14 Days

**DOI:** 10.1111/vec.13470

**Published:** 2026-03-20

**Authors:** Kate S. Farrell, Steven E. Epstein, Nghi Nguyen, Ronald H. L. Li

**Affiliations:** ^1^ Department of Veterinary Surgical and Radiological Sciences, School of Veterinary Medicine University of California, Davis Davis California USA; ^2^ Department of Clinical Sciences, College of Veterinary Medicine North Carolina State University Raleigh North Carolina USA

**Keywords:** aggregometry, blood bank, cold storage, storage lesions, thrombocytopenia, transfusion

## Abstract

**Objective:**

To assess platelet function, storage lesion development, and bacterial contamination in canine platelet concentrate (PC) units stored at 4°C for 14 days.

**Design:**

Prospective, in vitro experimental study.

**Setting:**

University teaching hospital.

**Animals:**

Six units of canine PC.

**Interventions:**

PC units were stored at 4°C for 14 days without agitation and sampled on Days 0, 5, 7, 9, 12, and 14. Testing included automated platelet count, platelet indices, WBC and RBC counts, blood gases, lactate, glucose, and electrolyte concentrations, light transmission aggregometry, alpha‐granule secretion, phosphatidylserine externalization via flow cytometry, and aerobic/anaerobic bacterial culture.

**Measurements and Main Results:**

Platelet count remained stable during storage, with median daily values >500,000/µL. Lactate concentration increased over time, with a median (range) value of 2.1 mmol/L (1.7–2.4 mmol/L) on Day 0 increasing to 9.6 mmol/L (8.1–10.3 mmol/L) on Day 14 (*p* < 0.0001). All pH measurements remained >7.1. While platelets remained viable throughout storage, platelet function varied over time and response was agonist dependent. Response to ADP was minimal on aggregometry, and alpha‐granule secretion increased on Days 12 and 14. Thrombin‐induced aggregation was higher compared to ADP and collagen until Day 12, but alpha‐granule secretion decreased after Day 7. Collagen failed to elicit robust aggregation response throughout the storage period, but activation with convulxin induced significant activation. Flow cytometry demonstrated variable P‐selectin and Annexin V expression in unstimulated and agonist‐treated platelets. Bacterial cultures revealed growth in one sample on Day 14.

**Conclusions:**

In canine PC units stored at 4°C for 14 days, there was evidence of loss of platelet function that was agonist dependent, but there were limited or expected changes in hematologic values, metabolic parameters, and platelet viability. Findings support further investigation of cold storage as an option for prolonging canine PC shelf life.

**Abbreviations**
AM: acetoxymethyl
FC: fold change
MFI: median fluorescence intensity
MPV: mean platelet volume
PAR: protease‐activated receptor
PC: platelet concentrate
PDW: platelet distribution width
PS: phosphatidylserine

## 1 Introduction

Platelet transfusions play a role in the management of patients with severe thrombocytopenia and, less commonly, thrombocytopathia in canine and human patients. While platelet concentrate (PC) has been recognized as the product of choice for controlling bleeding in these circumstances, supply is frequently limited by insufficient donations and a short shelf life [1, 2]. With substantial product loss due to expiration, few veterinary hospitals undertake the added cost and logistics of producing PC, leading to poor availability of a product to treat or prevent hemorrhage in patients with platelet abnormalities.

In human medicine, PC units are typically stored at room temperature (20–24°C) under gentle agitation for up to 5 days [3], though lifespan can be extended up to 7 days under restricted conditions [4]. Veterinary medicine has followed similar standards, with several studies demonstrating adequate platelet function, viability, and other parameters in PC units stored for 5–7 days at room temperature [5, 6, 7, 8]. The shelf life of PC has been limited to this duration primarily due to concerns about the potential for rapid proliferation of contaminating bacteria at room temperature [9], as well as a progressive decrease in platelet viability and function with time. The structural, biochemical, functional, and metabolic changes that platelets undergo during storage are referred to as platelet storage lesions, and this can result in reduced platelet function and half‐life after transfusion [10, 11, 12].

Given the limitations imposed by this short shelf life and concerns about the weak evidence supporting current storage practices, cold storage of PC (1–6°C) has been investigated in people to further extend PC shelf life and improve patient supply [13, 14, 15, 16]. Early concerns that platelets demonstrated reduced in vivo circulating half‐life to 1–2 days following cold storage (vs. 3–4 days with room temperature) led to a general abandonment of the practice [13–15, 17]. Yet, cold‐stored platelet units appear to have numerous advantages demonstrated in human studies, including inhibited growth of microorganisms [18], no requirement for agitation [19], adequate in vitro aggregation responses and metabolic activity [13, 15, 20], and potentially superior hemostatic efficacy [20, 21, 22, 23]. Recent in vivo studies in people using extended, cold‐stored platelets have also demonstrated analogous platelet recovery and survival compared to room‐temperature platelets [24] and adequate hemostasis [2].

In recent veterinary studies, canine PC showed minimal storage lesion development, maintenance of platelet function, and no bacterial growth when stored in plasma or additive solutions at 4°C for 7 days [25] and 21 days [26]. While these are important initial findings, the impact of cold storage over time on platelet integrin expression, viability, and agonist‐induced activation and aggregation remains unclear.

The objective of this study was to assess platelet viability, storage lesion development, bacterial contamination, and agonist‐induced platelet aggregation and activation in canine PC stored for 14 days at 4°C. We hypothesized that while cold storage of PC would induce an acceptable degree of storage lesions, it would not significantly impact platelet function and viability throughout the duration of storage compared to Day 0.

## 2 Methods

Client‐owned canine blood donors participating in the University of California, Davis, Canine Community Blood Donor Program were eligible for inclusion in this study. Dogs must meet standard criteria for enrollment in the blood donor program, which requires that donors be between 1 and 8 years old, >55 pounds (25 kg), in overall good health, and current on vaccines and flea, tick, and heartworm preventatives. Canine donors receive yearly physical examinations and bloodwork consistent with American College of Veterinary Internal Medicine guidelines [27]. The study was approved by the Institutional Animal Care and Use Committee (protocol #23450), and owner consent was acquired.

Standard practices used to acquire and process canine blood donations were employed for donations and blood processing. For each enrolled dog, fresh whole blood (approximately 450 mL) was collected into blood collection bags containing citrate phosphate dextrose anticoagulant^1^; the first 5 mL of blood was collected into a diversion sampling arm and discarded. Whole blood was processed into fresh PC as described below. Platelet‐rich plasma was generated by centrifugation of whole blood (1000 × *g*, 24°C) for 6 min and 45 s and isolated in a platelet‐specific satellite bag (XT‐612)^1^. Platelets were further concentrated via centrifugation (2000 × *g*, 24°C) for an additional 12 min. Supernatant was expressed into a transfer bag, leaving approximately 60 mL of plasma with a platelet pellet. This PC was kept at room temperature for 1 h and then gently agitated to resuspend the platelet pellet in the plasma. Units were stored in a refrigerator at 4°C without agitation for a total of 14 days.

On Days 0, 5, 7, 9, 12, and 14, 10 mL of sample was removed aseptically from each PC unit for testing. On Day 0, this was performed immediately after processing and prior to refrigeration. This sample served as room temperature control. On subsequent days, samples were removed from refrigeration and naturally reached room temperature prior to testing. To avoid repeated bag punctures and the risk of unit contamination, a sterile tube welding device was used for sampling^2^. Samples were analyzed with a hematology analyzer^3^ for platelet count, mean platelet volume (MPV), platelet distribution width (PDW), WBC count, and RBC count. To assess metabolic activity, a blood gas analyzer^4^ was used to measure pH, PO_2_, PCO_2_, bicarbonate, plasma lactate, glucose, ionized calcium, potassium, sodium, and chloride concentrations of each sample.

### 2.1 Platelet Viability Assay

**FIGURE 1:**
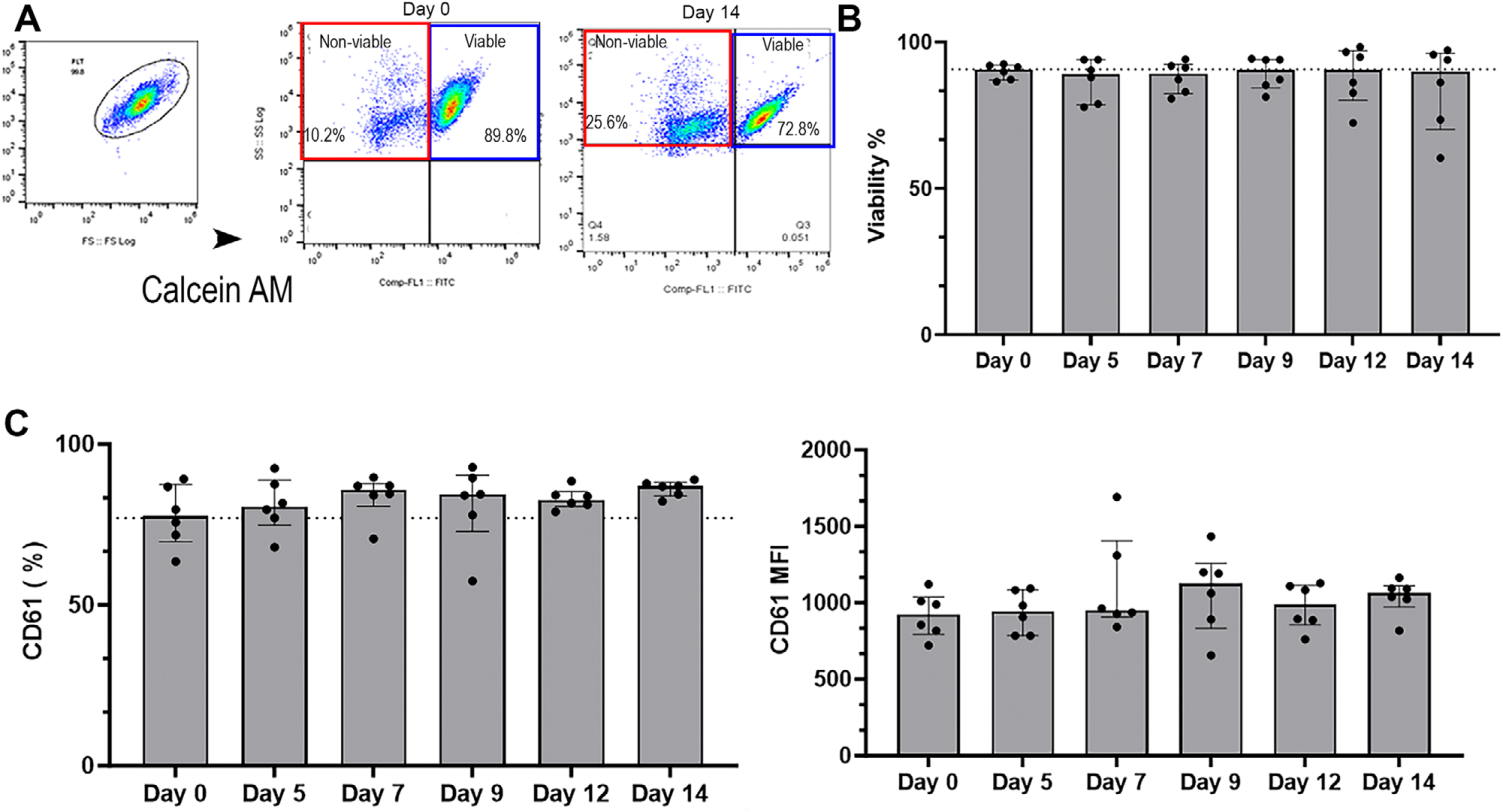
Assessment of platelet viability and integrin expression by flow cytometry of six canine platelet concentrate units stored at 4°C for 14 days. (A) Representative scatter dot plots illustrating forward‐ and side‐scatter properties of platelet concentrate from a blood donor after 14 days of storage. Platelets were loaded with Calcein acetoxymethyl (AM) ester, which was cleaved by intracellular esterases in viable cells, to yield a fluorescent signal (blue boxes). Nonviable platelets are shown in red boxes. (B) No statistically significant difference in platelet viability was detected over time. (C) Surface beta‐3 integrin on platelets was evaluated by immunolabeling of CD61. Integrin expression was measured as either percentage (%) positive or surface receptor density, expressed as median fluorescence intensity (MFI). No statistical changes were noted over time.

To assess platelet viability, PC was first standardized to 1 × 10^7^ cells/mL with Tyrode HEPES (pH 7.2, 5 mM dextrose without divalent cations), warmed to 37°C, and loaded with 2 µg/mL Calcein acetoxymethyl (AM) ester^5^ for 30 min at 37°C. Platelet metabolism of Calcein AM was halted by the addition of 5 mM EDTA before analysis by flow cytometry (excitation: 494 nm, emission: 517 nm). Only metabolically active platelets with intact cell membranes retain fluorescing Calcein AM, which was detected using flow cytometry (Figure 1A).

### 2.2 Assessment of Platelet Integrin Expression and Activation Response to Agonists

Flow cytometry analysis was performed to assess surface integrin expression, intrinsic platelet activation, and response to platelet agonists as previously described [28]. On each experimental day, PC was corrected to 1 × 10^7^ cells/mL with Tyrode HEPES (pH 7.2, 5 mM dextrose, no divalent cations) and warmed to 37°C in a bead bath for 30 min. Basal expressions of integrin beta‐3 and P‐selectin were detected by allophycocyanin‐conjugated mouse anti‐human monoclonal antibodies to CD61^6^ and fluorescein isothiocyanate‐conjugated rat anti‐mouse monoclonal antibody to CD62P^7^, respectively. Cross‐reactivity of monoclonal antibodies with canine platelets was evaluated and validated in previous studies [28, 29]. Samples were incubated for 45 min at 37°C before fixation with 1% methanol‐free formaldehyde.

To assess alpha‐granule secretion, PC samples were treated with 10 µM ADP^8^, 0.001 U/mL bovine α‐thrombin^9^, and 100 ng/mL convulxin^10^ for 10 min before immunolabeling of CD61 and CD62P as described above. Additionally, externalization of phosphatidylserine (PS) was assessed using fluorescein isothiocyanate‐conjugated Annexin V^11^ (1:200) after calcium loading. To avoid in vitro platelet activation, platelets were loaded with 1 mM CaCl_2_ every 15 min to a final concentration of 2 mM CaCl_2_. Basal expression of PS was assessed in resting (unstimulated) platelets. The capacity of PS externalization was induced by thrombin (0.001 U/mL, 15 min). Platelets treated with the calcium ionophore, A23187 (20 µM)^12^, served as positive controls. Platelets were then fixed in 1% methanol‐free formaldehyde and analyzed by flow cytometry.

### 2.3 Flow Cytometry Analysis

All samples were analyzed in a single batch using a five‐color flow cytometer^13^, and data were analyzed using commercially available software^14^. Platelets were identified using fluorophore‐conjugated antibodies to β3 integrin (CD61), and the platelet gate was established using fluorescence‐minus‐one controls, isotype controls, and 0.5–3 µm calibration beads as described [30]. Each sample was analyzed within the platelet gate until 10,000 events were recorded (Figure 1A). Fluorescence compensation was calculated using anti‐mouse compensation beads with mouse IgG isotype controls conjugated to the experimental fluorophore.

Activation response to each individual agonist was measured as median fluorescence intensity (MFI) based on P‐selectin expression in unstimulated (resting) and activated platelets on each day. Platelet responsiveness to agonists was calculated with the following formula:

$$\mathrm{MFI}\;\mathrm{fold}\;\mathrm{change}\;\left(\mathrm{FC}\right)=\log10\frac{\left(\mathrm{MFI}\;\mathrm{activated}\right)}{\left(\mathrm{MFI}\;\mathrm{resting}\right)}.$$

The degree of PS externalization in response to thrombin or A23187 was calculated using the following formula:

$$\begin{array}{ccc} & & \mathrm{Annexin}\;V\;\left(\%\;\mathrm{change}\right)=\\ & & \frac{\mathrm{Annexin}\;V\;\mathrm{activated}\;(\%\;\mathrm{positive})\;-\;\mathrm{Annexin}\;V\;\mathrm{resting}\;(\%\;\mathrm{positive})\;}{\mathrm{Annexin}\;V\;\mathrm{resting}\;(\%\;\mathrm{positive})}\\ & & \;\times \;100. \end{array}$$

### 2.4 Light Transmission Aggregometry

Platelet aggregation was measured by light transmission aggregometry^15^. Platelet‐poor plasma, generated by centrifugation of PC at 5000 × *g* for 15 min, and thrombin‐activated platelets served as positive aggregation control. PC was standardized to 1 × 10^8^ cells/mL using autologous platelet‐poor plasma in prewarmed siliconized cuvettes^16^ with magnetic stir bars set at 1600 rpm^17^. After 1 min of baseline measurement, ADP (10 µM)^8^, equine type I collagen (16 µg/mL)^18^, or bovine α‐thrombin (0.1 U/mL)^9^ were added, and aggregation was measured for an additional 5 min. Aggregation in response to agonists, expressed as maximum aggregation (% of aggregation) and slope, was calculated using commercially available software^19^. Final concentrations of agonists were determined by preliminary dose responses established in our laboratory and previously published studies to ensure >50% maximum aggregation in response to ADP and >80% maximum aggregation in response to collagen [31, 32].

### 2.5 Bacterial Culture

On each day, samples from every platelet unit were also added into separate aerobic^20^ and anaerobic^21^ blood collection bottles, allowing each unit to be cultured individually for every time point. Samples were submitted to the hospital's clinical microbiology laboratory for blood culture and bacterial identification under aerobic and anaerobic conditions. Briefly, this process included incubation of the blood in the blood culture collection bottles for 5 days. On Days 1, 2, and 5, an aliquot of blood was removed from the blood culture collection bottle and put onto solid agar for incubation under aerobic and anaerobic conditions. Any positive growth was identified for bacterial speciation.

### 2.6 Statistical Methods

All parameters on Days 5, 7, 9, 12, and 14 were compared to baseline (Day 0) to assess changes over the duration of storage. Given the sample size, data were assumed to be nonparametric and were presented as median (range). To determine the effect of time, a Friedman test was used to analyze values for each day compared to Day 0, with a post hoc Dunn's multiple comparisons test for pairwise comparisons. The response to platelet agonists on each testing day was compared using either the Wilcoxon test or the Friedman test, followed by post hoc Dunn's multiple comparisons tests, if multiple agonists were compared. Variability among donors was assessed by coefficient of variation as the ratio of the standard deviation to the mean. Significance differences between platelet units at Day 0 and subsequent days were determined when *p* < 0.05. Statistical analyses were performed using commercial statistical software^22^.

## 3 Results

### 3.1 Hematologic Parameters

Hematologic parameters for all days are presented in Table 1. Platelet count and WBC count did not differ significantly when comparing Day 0 to subsequent days. Median MPV was significantly increased on Day 5 (9.6 fL [8.2–10.4 fL], *p* = 0.003) and Day 14 (9.6 fL [7.3–10.2 fL], *p* = 0.035) compared to Day 0 (8.3 fL [7.7–9.7 fL]); however, Days 7, 9, and 12 did not differ from Day 0. Median PDW differed minimally but significantly on Day 14 (38.1% [35.2%–39.6%]) from Day 0 (36.2% [33.4%–38.5%]) (*p* = 0.022).

**TABLE 1:** Relevant hematologic parameters for canine platelet concentrate units (*n* = 6) stored at 4°C for up to 14 days.

	Day 0	Day 5	Day 7	Day 9	Day 12	Day 14
Platelet count (×10^9^/L or ×10^3^/µL)	648 (472–857)	517.5 (391–752)	597 (409–698)	564.5 (468–834)	548 (490–922)	602 (419–897)
Mean platelet volume (fL)	8.3 (7.7–9.7)	9.6 (8.2–10.4)^a^	8.2 (8.0–9.8)	8.9 (8.0–9.8)	9.4 (8.0–10.0)	9.6 (7.3–10.2)^b^
Platelet distribution width (%)	36.2 (33.4–38.5)	38.2 (34.1–40.0)	36.9 (34.1–39.3)	37.6 (35.2–38.7)	37.3 (34.8–39.3)	38.1 (35.2–39.6)^b^
WBC count (×10^9^/L or ×10^3^/µL)	0.76 (0.39–1.65)	1.17 (0.49–2.33)	0.80 (0.49–1.41)	0.65 (0.36–1.91)	0.58 (0.39–2.86)	0.63 (0.38–1.47)
RBC count (×10^6^/µL)	0.05 (0.04–0.08)	0.09 (0.05–0.10)^b^	0.07 (0.06–0.18)	0.07 (0.05–0.17)	0.07 (0.05–0.08)	0.07 (0.06–0.19)

*Note*: Data are presented as median (range).

^a^ Significance (*p* < 0.01) compared to Day 0.

^b^ Significance (*p* < 0.05) compared to Day 0.

### 3.2 Metabolic Parameters

Metabolic parameters are presented in Table 2. pH remained stable throughout most of the duration of storage, with the median value on Day 14 (7.252 [7.209–7.323]) being slightly higher than Day 0 (7.192 [7.136–7.217]) (*p* = 0.006). No single pH measurement was below 7.1. The PCO_2_ and bicarbonate concentration progressively decreased over time, with Days 9, 12, and 14 differing significantly from Day 0. Lactate concentration progressively increased over time, with a median value of 2.1 mmol/L (1.7–2.4 mmol/L) on Day 0 increasing to 9.6 mmol/L (8.1–10.3 mmol/L) on Day 14 (*p* < 0.0001). Median lactate concentration on Days 9, 12, and 14 was significantly higher than Day 0. The PO_2_ throughout storage did not differ from Day 0. The median value for glucose concentration remained above 27.8 mmol/L (500 mg/dL) on all days.

**TABLE 2:** Results of relevant metabolic parameters for canine platelet concentrate units (*n* = 6) stored at 4°C for up to 14 days.

	Day 0	Day 5	Day 7	Day 9	Day 12	Day 14
pH	7.192 (7.136–7.217)	7.217 (7.190–7.296)	7.230 (7.190–7.262)	7.246 (7.199–7.281)	7.257 (7.179–7.309)	7.252 (7.209–7.323)^a^
PO_2_ (mmHg)	107.5 (97.7–125.0)	118.5 (93.0–146.0)	99.0 (93.4–185.0)	126.5 (98.2–132.0)	110.5 (98.5–122.0)	119.0 (95.8–145.0)
PCO_2_ (mmHg)	40.1 (30.2–43.9)	29.2 (20.8–32.0)	25.6 (21.8–29.6)	22.3 (18.0–24.9)^b^	18.0 (16.7–20.7)^a^	14.7 (13.2–18.9)^c^
Bicarbonate (mmol/L)	14.6 (11.7–16.2)	11.4 (9.0–13.2)	10.2 (8.4–12.3)	9.1 (7.4–11.2)^b^	7.5 (6.3–9.2)^a^	6.6 (5.3–8.0)^c^
Lactate (mmol/L)	2.1 (1.7–2.4)	5.1 (4.4–5.4)	6.3 (5.9–6.6)	7.2 (6.6–7.7)^b^	8.6 (8.0–9.2)^d^	9.6 (8.1–10.3)^c^
Glucose (mmol/L)	30.2 (28.1–31.9)	28.7 (27.5–30.9)	29.3 (28.4–30.8)	29.1 (27.8–30.4)	28.2 (27.4–30.4)	27.8 (24.5–30.3)
Glucose (mg/dL)	544 (507–575)	517 (496–557)	528 (511–555)	524 (501–548)	508 (493–547)^b^	501 (442–546)^a^
Potassium (mmol/L)	2.7 (2.4–3.1)	3.3 (3.1–3.6)	3.3 (3.1–3.4)	3.3 (3.1–3.4)	3.3 (3.2–3.6)^a^	3.4 (3.2–3.6)^c^
Sodium (mmol/L)	159 (157–160)	159 (157–160)	158 (156–159)	158 (155–159)	159 (157–160)	159 (156–160)
Chloride (mmol/L)	86 (83–89)	87 (83–88)	87 (83–89)	87 (83–88)	86 (83–88)	88 (83–89)

*Note*: Data are presented as median (range).

^a^ Significance (*p* < 0.01) compared to Day 0.

^b^ Significance (*p* < 0.05) compared to Day 0.

^c^ Significance (*p* < 0.0001) compared to Day 0.

^d^ Significance (*p* < 0.001) compared to Day 0.

Potassium concentration differed slightly, with median values on Day 12 and 14 being increased compared to Day 0. Ionized calcium concentration was below the limits of detection at all time points.

### 3.3 Platelet Viability and Surface Integrin Expression

Representative flow cytometry dot plots of platelet viability are shown in Figure 1A. The median viability of PC was 90.65% (87.0%–92.5%) on Day 0. No significant difference in platelet viability was detected over the storage duration (Figure 1B). However, following the rewarming of PC on Days 12 and 14, there was increased variability among donors with coefficients of variation ranging between 11.3% and 17.4%, and median viability of 90.6% (72.4%–98.3%) and 90.1% (60.4%–97.2%), respectively.

Expression of integrin beta‐3 (CD61) was assessed throughout the study period. Integrin beta‐3 expression on the platelet surface assessed as percent positive or MFI did not differ during the storage period (Figure 1C).

### 3.4 P‐selectin Expression and Alpha Granule Secretion

**FIGURE 2:**
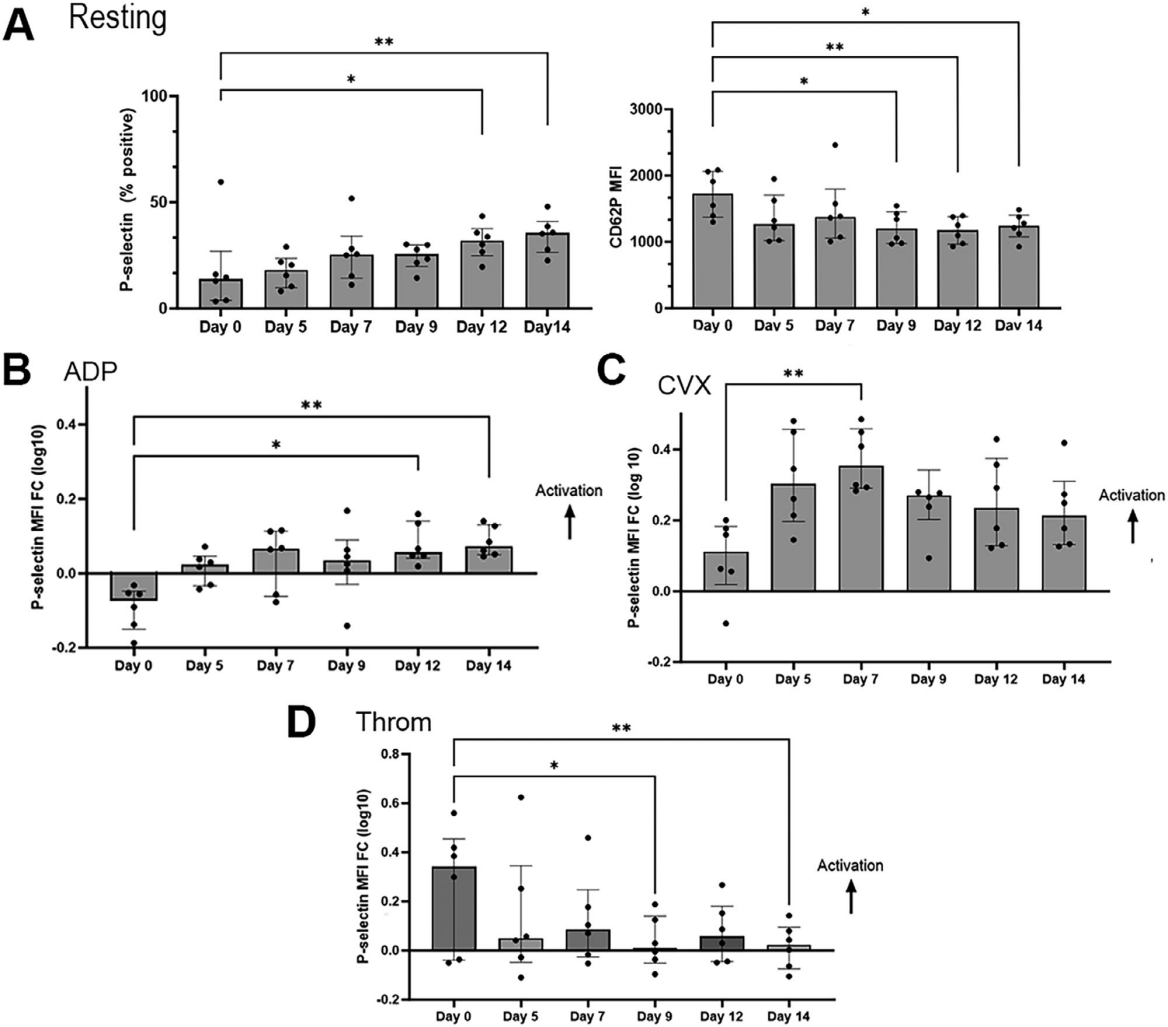
Flow cytometry analysis of platelet activation by surface P‐selectin expression (CD62P) in six canine platelet concentrate units stored at 4°C for 14 days. (A) In unstimulated (resting) platelets, platelet activation was measured as percentage of P‐selectin‐positive cells and P‐selectin surface density, measured as median fluorescence intensity (MFI). Over time, an increased number of P‐selectin‐positive platelets was detected and was significantly higher on Days 12 and 14. However, this did not translate to increased MFI over time since P‐selectin MFI was lower from Day 9. Platelet response to the agonists (B) adenosine diphosphate (ADP), (C) convulxin (CVX), and (D) thrombin (Throm) was measured by fold change (FC) in MFI (log10) relative to resting platelets. (B) Response to ADP was minimal until Days 12 and 14. (C) Compared to ADP, convulxin was able to elicit platelet activation throughout the storage period with maximum response noted on Day 7. (D) A decline in response to thrombin was noted. After Day 7, P‐selectin upregulation in response to thrombin was minimal and significantly less compared to Day 0.

Flow cytometry results are presented in Table S1. Platelet activation was assessed by evaluating P‐selectin (CD62P) expression in unstimulated (resting) (Figure 2A) and stimulated platelets (Figure 2B–D). In resting platelets, surface P‐selectin density, expressed as MFI, was significantly lower on Days 9, 12, and 14 compared to Day 0. Conversely, the number of platelets expressing P‐selectin was significantly higher on Days 12 and 14 compared to Day 0.

Platelet activation in response to agonists, measured as P‐selectin surface density compared to resting platelets (MFI FC), was variable over time depending on the agonist. In vitro stimulation of platelets with ADP (acting via P2Y_12_ and P2Y_1_ receptors) showed an increase in response on Days 12 and 14 compared to Day 0 (Figure 2B). Response to convulxin, a GPVI receptor agonist, expressed as increased P‐selectin density, was most profound on Day 7 compared to Day 0 (Figure 2C). When evaluating platelet response to protease‐activated receptor (PAR) stimulation using thrombin, the response was the highest on Day 0, then decreased thereafter with minimal responses noted on Days 9 and 14 (Figure 2D).

**FIGURE 3:**
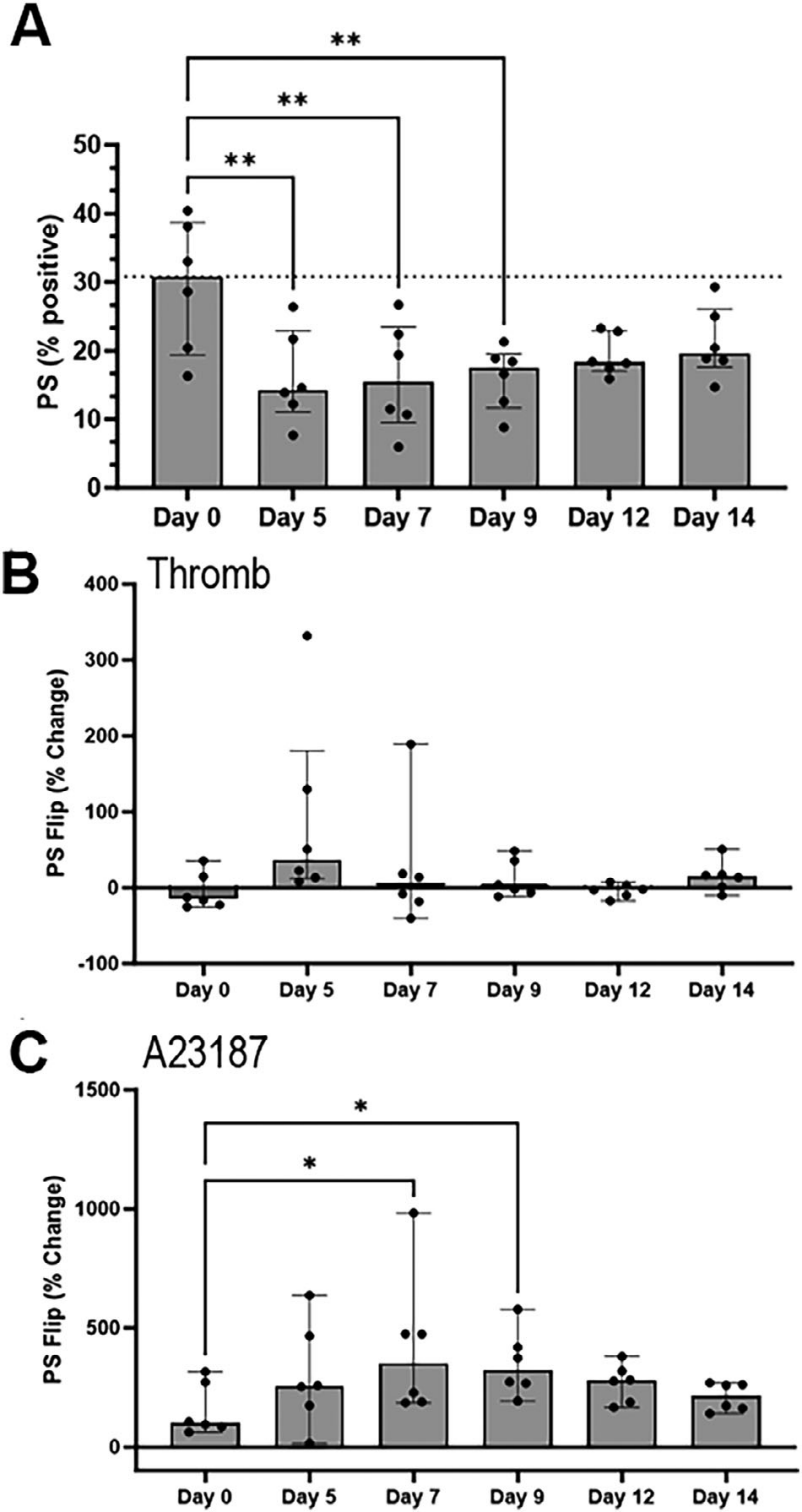
Flow cytometry analysis of phosphatidylserine (PS) externalization, detected on the platelet surface, in six canine platelet concentrate units stored at 4°C for 14 days. (A) In unstimulated platelets, the percentage of Annexin V‐positive platelets decreased on Days 5, 7, and 9 compared to Day 0. (B) To detect the ability of platelets to externalize PS, platelets were activated with thrombin (Thromb) in the presence of calcium. Percentage change in Annexin V compared to resting platelets (AV % change) remained static and close to zero throughout storage. (C) Compared to the positive controls of A23187‐treated platelets, PS externalization occurred throughout the storage period and increased on Days 7 and 9 from Day 0.

Externalization of PS on the platelet surface was assessed using Annexin V in resting and agonist‐treated platelets (Figure 3). In resting platelets, the percentage of platelets positive for PS was diminished on Days 5, 7, and 9 compared to Day 0; Days 12 and 14 did not differ from Day 0 (Figure 3A). In thrombin‐treated platelets, PS flip was low and remained static throughout storage (Figure 3B). On the contrary, A23187 was able to induce PS flip throughout the duration of the study, with the most profound externalization noted on Days 7 and 9 compared to Day 0 (Figure 3C).

### 3.5 Light Transmission Aggregometry

**FIGURE 4:**
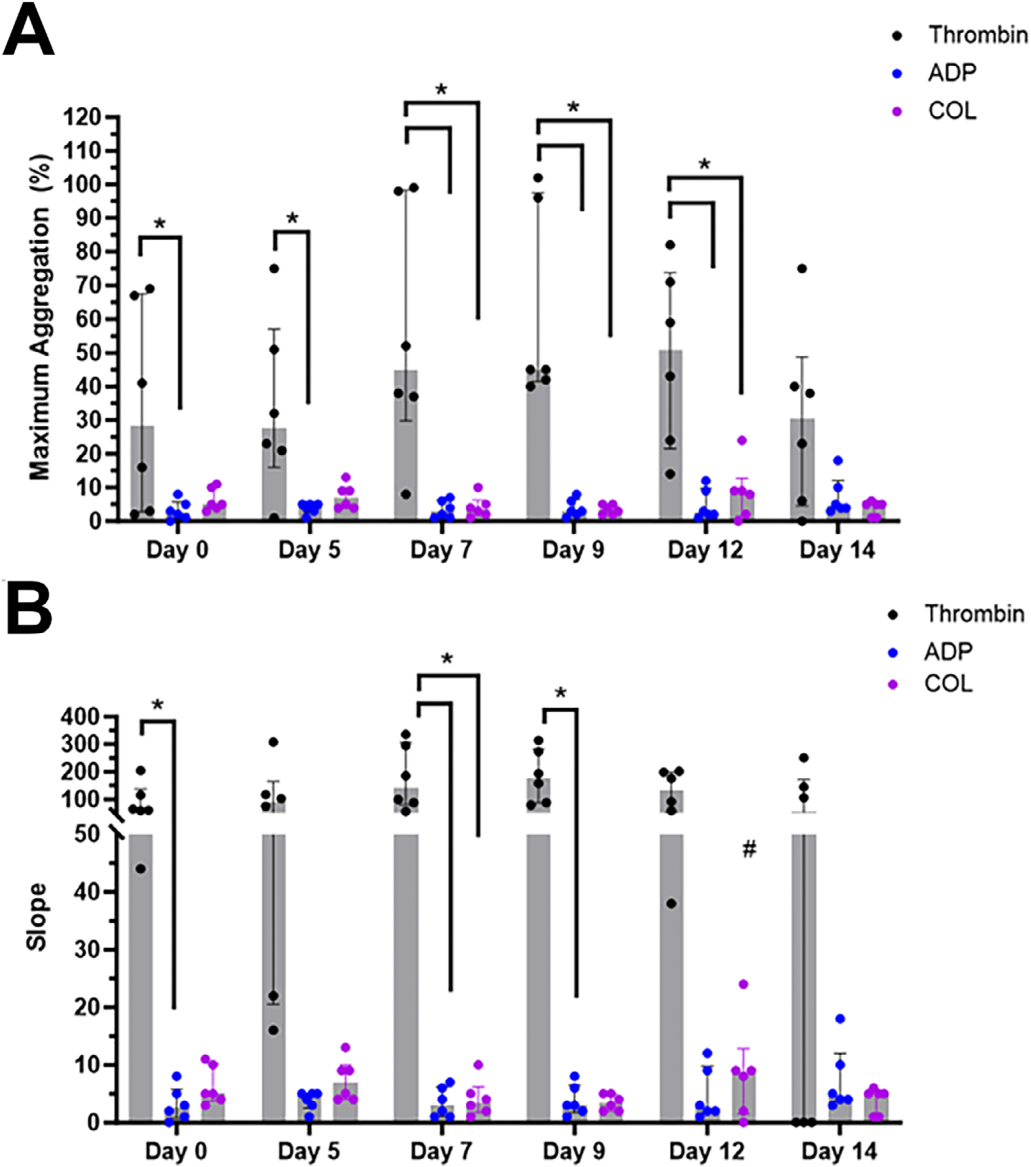
Light transmission aggregometry, evaluating platelet aggregation in response to thrombin, ADP, or collagen (COL) in canine platelet concentrate stored at 4°C for 14 days. Minimal aggregation response to ADP and COL in maximum aggregation (%) and slope was noted throughout the storage period, while response to thrombin was observed until Day 14.

Aggregometry results are presented in Table S2. In general, platelet aggregation, measured as maximum aggregation (%) and slope, in response to ADP remained low and did not change significantly throughout the storage period (*p* = 0.42 and *p* = 0.71, respectively) when compared to Day 0 (Figure 4). However, upon treatment of PC with collagen, aggregation (%) and slope increased over time (*p* = 0.034 and *p* = 0.0011, respectively). On Day 12, collagen‐induced aggregation was significantly increased compared to Day 0 (maximum aggregation: *p* = 0.04, slope: *p* = 0.027). Thrombin‐induced aggregation remained unchanged throughout the study period (maximum aggregation: *p* = 0.36, slope: *p* = 0.11). With the exception of Day 14, thrombin‐induced aggregation (% maximum aggregation) was significantly higher than ADP‐mediated aggregation (*p* < 0.05) throughout the storage period. Aggregation induced by thrombin was higher than collagen‐mediated aggregation on Days 7, 9, and 12 (*p* = 0.04, *p* = 0.028, and *p* = 0.04, respectively) (Figure 4A). When assessing the slope of the aggregation curves, thrombin resulted in a significantly greater slope compared to ADP and collagen on Day 7 (*p* = 0.03) (Figure 4B).

### 3.6 Bacterial Culture

All samples tested negative for aerobic and anaerobic bacterial growth with the exception of one unit on Day 14. This sample grew a fastidious gram‐positive cocci (*Granulicatella adiacens*) from subcultures on Days 2 and 5.

## 4 Discussion

In canine PC units stored at 4°C for 14 days, there was evidence of loss of platelet function and development of storage lesions such as increased lactate concentration over time. However, many of these storage lesions were mild or expected to occur during storage, and there was no difference in platelet viability over time. Findings support further investigation of cold‐stored canine platelets as an alternative to standard room‐temperature storage.

Platelet storage lesions are characterized by diminished function, viability, and metabolism of stored platelets compared to fresh platelets. Unfortunately, no single test available for clinical or research purposes is sufficient to characterize the changes that occur during storage or to predict hemostatic function and cell survival after transfusion [12, 33]. For that reason, a compilation of tests was utilized to evaluate platelet quality during storage, and several in vitro tests were applied in this study.

In cold‐stored canine PC units investigated here, minimal differences in hematologic parameters were found over time. Platelet count is one parameter that is dictated by human guidelines [34, 35], though standardized veterinary guidelines do not exist. Prior human and canine studies showed that platelet count may decline and platelet indices may change (including increases in MPV and PDW) due to cellular fragmentation, microvesiculation, activation, and aggregate formation that occur during storage [12, 36, 37]. In this study, platelet count did not vary over the 14 days of storage, with median values remaining over 500 × 10^9^/L (500,000/µL) on each day, similar to previous reports on cold‐stored canine PC [25, 26, 38]. Additionally, changes in MPV and PDW were clinically small and did not show meaningful trends in this study.

Of metabolic parameters assessed, the pH of PC units is also regulated by human guidelines, with the requirement that at least 90% of units sampled have a pH ≥6.2 at the end of allowable storage given the irreversible deterioration of platelets below this level [34, 35, 39–41]. Cold storage of human PC units has been shown to reduce changes in pH by limiting metabolic rates [15, 42, 43]. In this study, pH remained relatively static throughout storage, with median values of 7.19–7.26 and all measured values (7.14–7.32) satisfying human standards. In two canine studies, the pH of PC units in plasma remained more commonly in a 7.4–7.5 range during 7 and 21 days of storage at 4°C [25, 26]. Room temperature studies with canine PC in plasma have provided variable results, with two studies reporting mean pH values <6.2 on Day 5 of storage [5, 8].

Lactate accumulation is another common marker of platelet storage lesions, with glycolysis during storage resulting in concomitant lactate production and a decrease in pH [40]. Compared to room temperature storage, cold storage has been shown to significantly reduce lactate production and glucose consumption in human PC [15, 43–45]. In this study, lactate concentration progressively increased up to a median of 9.6 mmol/L during storage, while bicarbonate concentration decreased. A range of lactate concentrations have been reported for canine PC stored at 4°C (>3 mmol/L on Day 7 [25]; 8.6 mmol/L on Day 21 [26]) and 22°C (2.8 mmol/L on Day 5 [5]; >10 mmol/L on Day 7 [7]). A study directly comparing room temperature and cold‐stored canine platelets is needed for further comparison.

While an overall decrease in platelet viability was not detected throughout the storage period, an increased variation in platelet viability on Days 12 and 14 was noted. We hypothesized that a portion of stored platelets might have undergone some form of cell death during storage. In human platelets, loss of mitochondrial membrane potential and apoptosis can be detected as soon as 4 days after cold storage [46]. Although the mechanisms of cold‐induced apoptosis in platelets are not well understood, the clustering of the platelet receptor, GPIb‐α, caused by binding to von Willebrand factor or a decrease in membrane fluidity may play a role in activating the intrinsic apoptotic pathways [46, 47]. This is supported by the marked externalization of PS upon treatment with exogenous calcium and the calcium ionophore A23187 starting from Days 7 and 9. Influx of calcium could exacerbate existing mitochondrial injury and increase reactive oxygen species, resulting in PS externalization via apoptosis or further intracellular hypercalcemia [48, 49].

The functional consequences of cold storage were tested by evaluating the various hemostatic functions of platelets such as degranulation, PS externalization, and aggregation using several physiologic agonists. While cold‐induced activation of platelets occurred during the storage period as shown by an increased number of platelets with P‐selectin, the surface density of P‐selectin, measured as MFI, decreased over time. This can occur due to the lipid phase transition of the platelet membrane, causing morphologic shape changes and increases in intracellular calcium in the absence of exogenous calcium [50]. Interestingly, the decrease in P‐selectin density could be secondary to shedding of P‐selectin caused by proteolytic cleavage [51].

Thrombin induces a strong stimulatory response in platelets by cleaving PARs, causing robust aggregation and enhanced procoagulant potential by externalizing electronegative phospholipids like PS to facilitate activation of clotting factors and further thrombin formation. Storage of human PC at room temperature has been shown to have downregulation in PAR1 and PAR4 by Day 6 of storage as a result of proteolysis, thereby reducing platelet aggregation and thrombin generation [52]. Similarly, we found that cold storage modulated thrombin‐mediated platelet activation, aggregation, and PS flip throughout the storage period. Thrombin is known to induce robust aggregation causing nearly maximum aggregation [53]. However, this was reduced nearly by half throughout the storage period. As platelet responsiveness to thrombin is crucial for primary hemostasis and thrombin generation during secondary hemostasis, future studies should investigate whether cold‐stored platelets could maintain adequate thrombin generation despite a loss in response to thrombin.

Of the agonists tested, responsiveness to ADP was severely affected throughout the storage period. One plausible explanation is that the processing of platelets could lead to intracellular ADP depletion via dense granule release. ADP‐mediated activation and aggregation rely on ADP via a paracrine and autocrine fashion. Thus, premature dense granule release could result in refractoriness to ADP stimulation in PC. A study in human PC showed that the addition of an exogenous ADP analog with ADPase apyrase, which inhibits ADP degradation, was able to preserve ADP‐induced aggregation and thrombus formation [54]. The function of two ADP receptors, P2Y_1_ and P2X_1_, could also rapidly deteriorate at 4°C, while the function of P2Y_12_ is preserved [55]. Canine platelets are not only less responsive to ADP compared to other species, but their ADP‐mediated activation is two to three times more dependent on P2Y_1_ compared to human platelets [56]. Future studies should measure dense granule release following platelet processing and the addition of anticoagulants.

The function of platelet glycoprotein VI (GPVI) in cold‐stored platelets was evaluated by in vitro treatments of collagen and the potent GPVI agonist, convulxin. Similar to ADP, collagen‐induced aggregation was minimal, while convulxin induced robust alpha granule secretion throughout the storage period. This level of modulation in collagen responsiveness is consistent with findings from a human study, which found a significant decrease in aggregation to collagen after the transfusion of cold‐stored platelets to human subjects [57]. The same investigators attributed this observation to the decrease in GPVI expression found only in platelets stored at 4°C. Since convulxin has a much higher affinity to GPVI than collagen, we could detect persistent activation when platelets were treated with convulxin, while the response to collagen was modulated likely due to GPVI downregulation [58]. Despite these lesions, cold‐stored human platelets were able to form larger aggregates than platelets stored at room temperature when they were subjected to high shear in collagen‐coated microchannels [57]. This suggests that the loss of GPVI function may be negligible in vivo.

All samples tested negative for aerobic and anaerobic bacterial growth with the exception of one unit on Day 14. While this may be consistent with a contaminant grown in the laboratory after removal from the platelet unit, true bacterial contamination of the unit must be considered. *Granulicatella adiacens* is a rarely identified fastidious gram‐positive cocci, mostly reported in cases of bacteremia and infective endocarditis in people [59]. To the authors' knowledge, it has not been identified as a PC contaminant in prior human studies. In general, transfusion‐transmitted reactions arising from bacterial contamination of blood components—and particularly PC stored at room temperature—are well documented in human medicine, leading to considerable morbidity and mortality [9, 60, 61]. Bacterial contamination of units in veterinary medicine is reported less frequently, likely due to the uncommon use of fresh platelet products; however, transfusion consequences may be severe and include cardiovascular collapse and death [62]. A major advantage of cold storage has been its demonstrated ability to slow the growth of bacteria, which allows for the prolongation of shelf life and a reduced risk of transfusion‐related sepsis [18]. Additional large studies may be required to determine the true incidence of bacterial contamination in canine cold‐stored PC.

There are several limitations to this study. For one, the small sample size increases the likelihood of type II errors. The high interindividual variability in platelet function among canine donors may also contribute to inaccuracies. While cold‐stored PC units were compared to Day 0 units (at room temperature prior to refrigeration) in this study, we did not include controls consisting of stored room‐temperature PC. With room temperature storage for 5–7 days being the current standard, direct comparisons would provide more insight into the loss of platelet function when platelets were stored at different temperatures. Multiple parameters were evaluated in this study to investigate platelet storage lesion development, but all potential in vitro changes could not be assessed. In addition, while in vitro testing is necessary prior to in vivo evaluation, controversy remains regarding the ability of in vitro testing to completely reflect in vivo platelet function, survival, and hemostatic capabilities. Lastly, our viability assay by flow cytometry only assessed intact platelets with the metabolic capacity of esterase‐mediated cleavage of Calcein and did not evaluate platelet lysis caused by storage over time.

In conclusion, storage of canine PC units at 4°C minimized hematologic and metabolic derangements that can be seen with prolonged platelet storage. While there was evidence of agonist‐dependent variability and loss of platelet function, platelets remained viable throughout 14 days of storage. Given the limitations of room‐temperature PC storage and emerging evidence for potential advantages of cold storage, further in vitro and in vivo studies of cold‐stored platelets are warranted to improve the shelf life and safety of canine PC.

## Ethics Statement

The authors confirm that the ethical policies of the Journal, as noted on the Journal's author guidelines page, have been adhered to and the appropriate ethical review committee approval has been received. The study was approved by the University of California, Davis Institutional Animal Care and Use Committee (protocol #23450), and owner consent was acquired.

## Conflicts of Interest

Dr. Epstein is an Assistant Editor of the Journal but only participated in the peer review process as an author. No other conflicts of interest are declared.

[Correction added on 10 April 2026, after first online publication: “Conflicts of Interest” has been updated.]

## Supporting information




**Supplemental Table 1**: Flow cytometry results for canine platelet concentrate units (*n* = 6) stored at 4°C for up to 14 days.
**Supplemental Table 2**: Light transmission aggregometry results for canine platelet concentrate units (*n* = 6) stored at 4°C for up to 14 days.

## Endnotes

1 Imuflex WB‐SP Blood Bag System, Terumo BCT Inc., Lakewood, CO.

2 Samplok sampling kit, ITL Biomedical, Reston, VA.

3 HM5, Zoetis, Parsippasny‐Troy Hills, NJ.

4 ABL827 FLEX, Radiometer, Brønshøj, Denmark.

5 Calcein AM Viability Dye, Thermo Fisher Scientific, Waltham, MA.

6 1:1000, Clone: VI‐PL2, eBioscience, San Diego, CA.

7 1:200, Clone: RB40.34, BD Pharmingen, San Jose, CA.

8 Adenosine diphosphate, Millepore Sigma, Burlington, MA.

9 Bovine α‐thrombin, Prolytix, Essex Junction, VT.

10 Convulxin, Cayman Chemical, Ann Arbor, MI.

11 Annexin V, Thermo Fisher Scientific INC, Thermo Fisher Scientific, Waltham, MA.

12 A23187, Millepore Sigma, Burlington, MA.

13 FC500, Beckman Coulter, Miami, FL.

14 Flowjo 10.0, BD, Ashland, OR.

15 Chronolog 700, Chronolog Corporation, Havertown, PA.

16 Cuvette, Chronolog Corporation, Havertown, PA.

17 Stir bar, Chronolog Corporation, Havertown, PA.

18 Collagen, Chronolog Corporation, Havertown, PA.

19 AGGRO/LINK, Chronolog Corporation, Havertown, PA.

20 BD BACTEC Pediatric aerobic bottle, Franklin Lakes, NJ.

21 BD BACTEC anaerobic bottle, Franklin Lakes, NJ.

22 Graphpad Prism version 10, GraphPad Software, La Jolla, CA.
