# Supplementary material for: Assessment of Platelet Storage Lesions, Viability, and Function in Canine Platelet Concentrate Units Stored at 4°C for 14 Days
Source: J Vet Emerg Crit Care (San Antonio). 2026 Mar 20;36(2):177–88. doi: 10.1111/vec.13470 (PMC13150405; doi:10.1111/vec.13470)
Supplement: Supplementary file 1 — Supplemental Table 1: Flow cytometry results for canine platelet concentrate units (n = 6) stored at 4°C for up to 14 days. Supplemental Table 2: Light transmission aggregometry results for canine platelet concentrate units (n = 6) stored at 4°C for up to 14 days. [file VEC-36-177-s001.docx]

**Supplemental Table 1.** Flow cytometry results for canine platelet concentrate units (n=6) stored at 4°C for up to 14 days.

| **Markers** |  | **Day 0** | **Day 5** | **Day 7** | **Day 9** | **Day 12** | **Day 14** |
| --- | --- | --- | --- | --- | --- | --- | --- |
| **CD61 (%)** |  | 77.7 (63.5-89.2) | 80.7 (68.0-92.5) | 85.8 (70.6-89.7) | 84.3 (57.5-92.9) | 82.8 (79.0-88.5) | 87.0 (82.3-89.0) |
|  | **Agonists** |  |  |  |  |  |  |
| **P-selectin**  **(MFI)** | **None (resting)** | 1731  (1301-2086) | 1268 (1010-1950) | 1375 (1006-2461) | 1200 (967-1545)^*^ | 1177 (931-1396)^§^ | 1246 (928-1484)^*^ |
| **P-selectin**  **(MFI FC [log 10])** | **ADP** | -0.073  (-0.188 – -0.032) | 0.023  (-0.043 – 0.072) | 0.066  (-0.078 – 0.116) | 0.035  (-0.141 – 0.168) | 0.057  (0.019 – 0.160)^*^ | 0.073  (0.045 – 0.140)^§^ |
|  | **Thrombin** | 0.341  (-0.050 – 0.560) | 0.049  (-0.110 – 0.624) | 0.087  (-0.053 – 0.459) | 0.012  (-0.097 – 0.188)^*^ | 0.058  (-0.049 – 0.267) | 0.023  (-0.106 – 0.142)^§^ |
|  | **Convulxin** | 0.111  (-0.091 – 0.201) | 0.303  (0.145 – 0.481) | 0.354  (0.283 – 0.486)^§^ | 0.271  (0.094 – 0.530) | 0.235  (0.122 – 0.429) | 0.213  (0.126 – 0.419) |
| **PS (%)** | **None (resting)** | 30.80 (16.30-40.40) | 14.25 (7.66-26.40)^§^ | 15.45 (5.95-26.70)^§^ | 17.50 (8.80-21.30)^§^ | 18.30 (15.90-23.30) | 19.65 (14.70-29.30) |
| **PS**  **(% change)** | **Thrombin** | -14.55  (-25.25 – 35.58) | 36.70  (8.33 –331.7) | 2.94  (-40.21– 189.1) | 1.23  (-11.64 – 48.41) | -2.32  (-17.17 – 7.61) | 15.34  (-9.93– 51.02) |
|  | **A23187** | 99.9 (62.1-316.0) | 255.5 (15.1-636.3) | 351.1 (185.8-982.4)^*^ | 323.5 (192.9-578.4)^*^ | 279.4 (166.1-380.5) | 215.7 (140.2-269.9) |
| **Calcein-AM**  **(%)** | **None** | 90.7 (86.5-92.5) | 89.2 (77.8-94.0) | 89.3 (80.7-94.1) | 90.6 (81.3-94.3) | 90.6 (72.4-98.3) | 90.1 (60.4-97.2) |

*Note:* Data is presented as median (range).

*Abbreviations:* FC, fold change; MFI, median fluorescence intensity; PS, phosphatidylserine

^*^ Denotes significance (*P* < 0.05) compared to Day 0.

^§^ Denotes significance (*P* < 0.01) compared to Day 0.

**Supplemental Table 2.** Light transmission aggregometry results for canine platelet concentrate units (n=6) stored at 4°C for up to 14 days.

|  | **Agonist** | **Day 0** | **Day 5** | **Day 7** | **Day 9** | **Day 12** | **Day 14** |
| --- | --- | --- | --- | --- | --- | --- | --- |
|  |  |  |  |  |  |  |  |
|  | **ADP** |  |  |  |  |  |  |
| **Max aggregation (%)** |  | 2.5 (0.75 – 5.75) | 4.5 (2.5 – 5.0) | 3.0 (1.0 – 6.25) | 3.0 (1.75 – 6.5) | 2.5 (1.75 – 9.75) | 4.5 (3.75 – 12.0) |
| **Slope** |  | 19.0 (11.25 – 45.5) | 21.5 (6.5 – 64.5) | 12.5 (4.5 – 25.5) | 24.0 (9.5 – 49.5) | 17.0 (9.0 – 28.25) | 10.0 (6.5 – 49.0) |
|  | **Collagen** |  |  |  |  |  |  |
| **Max aggregation (%)** |  | 5.0 (3.75 – 10.25) | 7.0 (4.0 – 15.5) | 3.5 (1.75 – 6.25) | 3.5 (2.0 – 5.0) | 6.0 (1.5 – 12.75) | 5.0  (1.0 – 5.25) |
| **Slope** |  | 64.0 (50.25 – 68.75) | 53.5 (41 – 67.75) | 13.5 (6.5 – 30.75) | 11.5 (4.5 – 27.75) | 8.0 (3.0 – 54.5) | 9.0 (7.0 – 34.75) |
|  | **Thrombin** |  |  |  |  |  |  |
| **Max aggregation (%)** |  | 28.5 (2.75 – 67.5) | 27.5 (16.0 – 57.0) | 45.0 (29.75 – 98.25) | 45.0 (41.5 – 97.5) | 51.0 (21.5 – 73.75) | 30.5 (4.5 – 48.75) |
| **Slope** |  | 62.0 (2.75 – 67.5) | 88.5 (20.5 – 164.8) | 142.0 (80.0 – 305.3) | 175.5 (85.75 – 283.3) | 64.0 (17.5 – 99.75) | 53.0 (0 – 171.5) |
